# Potential of Near-Infrared Spectroscopy (NIRS) for Efficient Classification Based on Postharvest Storage Time, Cultivar and Maturity in Coconut Water

**DOI:** 10.3390/foods12122415

**Published:** 2023-06-20

**Authors:** Xiaojun Shen, Tao Wang, Jingyi Wei, Xin Li, Fuming Deng, Xiaoqing Niu, Yuanyuan Wang, Jintao Kan, Weimin Zhang, Yong-Huan Yun, Fusheng Chen

**Affiliations:** 1 Coconut Research Institute, Chinese Academy of Tropical Agricultural Sciences, Wenchang 571339, China; 2 College of Food Science and Technology, Huazhong Agricultural University, Wuhan 430070, China; 3 School of Food Science and Technology, Hainan University, Haikou 570228, China; 4 The Innovation Platform for Academicians of Hainan Province, Wenchang 571339, China

**Keywords:** *Cocos nucifera* L., liquid endosperm, dwarfs, non-destructive analysis, discrimination

## Abstract

Coconut water (CW) is a popular and healthful beverage, and ensuring its quality is crucial for consumer satisfaction. This study aimed to explore the potential of near-infrared spectroscopy (NIRS) and chemometric methods for analyzing CW quality and distinguishing samples based on postharvest storage time, cultivar, and maturity. CW from nuts of Wenye No. 2 and Wenye No. 4 cultivars in China, with varying postharvest storage time and maturities, were subjected to NIRS analysis. Partial least squares regression (PLSR) models were developed to predict reducing sugar and soluble sugar contents, revealing moderate applicability but lacking accuracy, with the residual prediction deviation (RPD) values ranging from 1.54 to 1.83. Models for TSS, pH, and TSS/pH exhibited poor performance with RPD values below 1.4, indicating limited predictability. However, the study achieved a total correct classification rate exceeding 95% through orthogonal partial least squares discriminant analysis (OPLS-DA) models, effectively discriminating CW samples based on postharvest storage time, cultivar, and maturity. These findings highlight the potential of NIRS combined with appropriate chemometric methods as a valuable tool for analyzing CW quality and efficiently distinguishing samples. NIRS and chemometric techniques enhance quality control in coconut water, ensuring consumer satisfaction and product integrity.

## 1. Introduction

Tender coconut water (CW), the main edible part from the nuts of *Cocos nucifera* L., has gained immense popularity as a health drink due to its pleasant flavor [1], rich nutritional components [2], and therapeutic properties [2,3]. However, the organoleptic and functional characteristics of CW can vary based on factors, such as postharvest storage time, coconut cultivar, and maturity [4,5], posing challenges for industrial producers in utilizing it as raw material. 

Time to maturity for *Cocos nucifera* L. nuts typically takes around 11–12 months, and it can be classified into three stages: immature or tender (6–8 months), mature (9–11 months), and overly-mature (12 months or older) [6]. CW from nuts around 8 months is generally preferred as a refreshing drink due to its delicate flavor, while that from younger fruits tastes sour, and older ones have an unpleasant taste [4]. However, there is currently no efficient method to differentiate between the different developmental stages of CW.

*Cocos nucifera* L. can be categorized into two main groups: tall and dwarf. While tall is the predominant cultivar worldwide, dwarfs have gained attention in recent years, particularly for CW production due to the superior taste [7]. In China, the Wenye series of Dwarf cultivars (Wenye No. 2–6) has become popular among growers for the high yield, early production, and sweet water content. Among these cultivars, Wenye No. 2 is selected from ‘Malaya Yellowing Dwarf’ and is distinguished by its yellow-colored fruit, while Wenye No.4 is screened from an aromatic coconut known for its sweet juice rich in 2-acetyl-1-pyrroline (2AP). However, based on our knowledge, there has been no investigation into the efficient differentiation of CW from different cultivars. 

Postharvest conditions can have a detrimental effect on CW quality. The initial indicator of quality decline is the deterioration in organoleptic properties, mainly caused by changes in metabolites, such as amino acids, organic acids, sugars, phenolics, and nucleotides [5]. Off-flavors in the endosperm of postharvest coconuts can also arise from the metabolism of β-oxidation and lipoxygenase pathways [8,9]. Therefore, accurately and quickly determining the freshness of CW is crucial for drink manufacturers, but it remains a challenge.

Sugar and acid quality significantly affect the flavor of CW, making it important to monitor these constituents for product quality. However, conventional methods for analysis are often time-consuming and expensive. Near-infrared spectroscopy (NIRS) is a non-destructive and efficient method for measuring various constituents in different commodities. It has been successfully applied to evaluate various aspects of coconut products, including fat content [10], soluble solids [11], salt content [12], and pH value [13], in curry soup containing coconut milk. Moreover, NIRS has shown promising results in assessing the total soluble sugar content of coconut kernels from different cultivars and maturities [14]. However, the application of NIRS in detecting sugar and acid quality in CW, as well as discriminating CW based on postharvest storage time, cultivar, and maturity, has not been reported. Therefore, this study aims to address the gap by evaluating CW quality parameters and exploring the potential of NIRS combined with chemometric analysis. Further research in this area could potentially provide a rapid and cost-effective means of monitoring CW quality during production and handling.

Taking CW from coconuts of different storage time, coconut cultivars, and maturities as the research object, we evaluated the quality parameters (TSS, pH, TSS/pH, reducing sugar content, and soluble sugar content) of CW in postharvest coconut stored at 25 °C and collected NIRS information for each sample, intended to: (1) develop a calibration model to predict the quality parameter using NIRS as a rapid and nondestructive tool; (2) explore the possibility of using NIRS combined with chemometric analysis to discriminate CW according to postharvest storage time, coconut cultivar, and maturity. The results of this study will contribute to the understanding of coconut water analysis, provide valuable insights for quality control, and offer a basis for further research in this field. 

## 2. Materials and Methods

### 2.1. Sample Treatments

Coconuts of ‘Wenye No. 2’ and ‘Wenye No. 4’ cultivars were obtained from Coconut Germplasm Repository of Ministry of Agriculture and Rural Affairs in Wenchang, China. The coconuts were harvested in November 2020, with ‘Wenye No. 2’ coconuts collected at 8-month (No. 2-8M) and 10-month (No. 2-10M) maturity stages, and ‘Wenye No. 4’ coconuts collected at 6 months (No. 4-6M) and 8 months (No. 4-8M). After harvesting, coconuts were immediately transported to the laboratory of Coconut Research Institute and stored at 25 °C until measurement. Eliminating the fruits with physiological disorders, infections, or mechanical damage, a total of 544 coconuts with uniform shape were obtained, including 198 coconuts from No. 2-8M, 77 coconuts from No. 2-10M, 63 coconuts from No. 4-6M, and 206 coconuts from No. 4-8M. Each day, three coconut samples from No. 2-10M and No. 4-6M and five coconuts from No. 2-8M and No. 4-8M were randomly selected and individually measured. Total soluble solids (TSS) and pH of CW were immediately measured upon extraction. Additionally, samples were frozen in liquid nitrogen and stored at −80 °C for further analysis, including NIRS profiling collection, reducing sugar content, and soluble sugar content.

### 2.2. Measurement of Biochemical Properties

Biochemical analysis was conducted to determine the TSS, pH, reducing sugar content, and soluble sugar content. TSS was measured using a hand-held digital refractometer (ATAGO PAL-1, Tokyo, Japan). This instrument was zero-set before use, and an appropriate amount of the sample was transferred to the prism using a disposable dropper. The measurement was recorded accordingly. The pH value was measured using a METTLER TOLEDO FE20 pH meter, calibrated with standard buffers (pH 4.01, 7.0 and 9.21) prior to measurement. The pH meter’s probe was immersed in the sample, and the measurement was recorded. The sugar acid ratio, calculated as TSS/pH value, was determined according to the method described by Chen et al. [15]. 

The contents of soluble sugar and reducing sugar were carried out according to the method described by Shen et al. [5]. In this method, 1 g of coconut water was mixed with 3 mL of a potassium ferrocyanide solution (10.6 g mL^−1^) and 3 mL of a zinc acetate solution (21.9 g mL^−1^). The resulting mixture was then diluted to a volume of 50 mL and filtered. To determine the content of reducing sugar, 0.5 mL of the filtered solution was combined with 1.5 mL of distilled water and 4 mL of a 3,5-dinitrosalicylic acid solution. The mixture was then heated in a boiling water bath for 5 min and subsequently diluted to a final volume of 10 mL. After cooling, the solution was measured at a wavelength of 540 nm using an ultraviolet-VIS spectrophotometer (ultraviolet-1600, Shanghai Aoyi, Shanghai, China). For the determination of soluble sugar, 10 mL of the filtered solution was mixed with 1 mL of hydrochloric acid solution (6 mol L^−1^) and heated at a temperature of 80 °C for 10 min. After cooling, methyl red was added as an indicator, and sodium hydroxide solution (6 mol L^−1^) was added until the mixture turned light orange in color. The resulting mixture was then diluted to a final volume of 25 mL and reacted with 3,5-dinitrosalicylic acid. The absorbance of the solution was measured at a wavelength of 540 nm using the ultraviolet-VIS spectrophotometer. The total soluble sugar content was calculated based on the obtained absorbance, using a standard curve generated from a glucose standard solution. The content of soluble sugar was expressed as a percentage.

### 2.3. NIRS Measurement 

NIRS analysis was conducted using an NIR-Flex N500 model (Buchi Labortechnik AG, Switzerland) to acquire transreflectance spectra in a wavelength range of 1000–2500 nm (10,000–4000 cm^−1^) at a spectral resolution of 4 cm^−1^. Each spectrum contained 1501 data points. The spectrometer was equipped with a tungsten halogen lamp and a temperature-controlled extended range Indium–Gallium–Arsenide (InGaAs) detector. A 2 m long fiber optic probe with two embedded fiber bundles, one with a diameter of 2.0 mm for the light beam and one with a diameter of 3.5 mm for the light collector, was used. Prior to measurement, samples were equilibrated to room temperature using a water bath and analyzed on the same day. Each sample (1 mL) was placed in an 8 mm PE-stopfen flacon and analyzed three times without repositioning under thermostatic condition at 35 °C. Each spectrum acquisition consisted of 32 single scans. The acquired spectral data were documented in transmission (T) units and converted to absorbance (A) units (log (1/T)). Three received spectra, each consisting of 1131 data points after eliminating abnormal bands, were averaged to obtain a single spectrum for quantitative and qualitative analysis. 

### 2.4. Chemometrics Analysis and Data Analysis 

The partial least squares regression (PLSR) approach was employed to establish a relationship between the spectra and biochemical reference values using 544 CW samples. The Monte Carlo Sampling method was utilized to remove outliers [16]. The preprocessed spectra were divided into a training set and an external validation set at an approximate ratio of 3:1. The spectra were preprocessed using smoothing and the second derivative (2nd Der) algorithms. Three wavelength selection algorithms, including competitive adaptive reweighting sampling (CARS) [16], variable combination population analysis (VCPA) [17], and interval combination optimization (ICO) [18], were used to select key variables to optimize the model. The root mean squared error of cross-validation was obtained using 5-fold cross-validation method, and the minimum value served as a reference parameter to determine the optimal number of latent variables.

The performance of the quantitative model equations was evaluated using several metrics, including the coefficient of determination for calibration ($R_{C}^{2}$) and prediction ($R_{P}^{2}$), the root mean squared error of fitting (RMSEF) and prediction (RMSEP), and the residual prediction deviation (RPD). A good model should have higher R^2^ and RPD values and lower RMSE. The RPD was classified as proposed by Chang et al. [19]: (A) excellent models (RPD > 2.0); (B) useful models (1.4 < RPD < 2.0); (C) unreliable models (RPD < 1.4). The R^2^ values were classified into 5 levels according to Saeys et al. [20]: (A) poor predictions (R^2^ < 0.50); (B) moderate predictions (0.50 < R^2^ < 0.65); (C) approximate quantitative predictions (0.66 < R^2^ < 0.81); (D) good quantitative prediction (0.82 < R^2^ < 0.90); (E) excellent prediction (R^2^ > 0.91). All these analyses were conducted using MATLAB 7.10.0 (R2010a) (Math Works Inc., Natick, MA, USA) with the libPLS toolbox (Version 1.98).

Discriminant analysis was performed using SIMCA-P v14.1 software (Umetrics, Umeå, Sweden) to distinguish samples based on three matrices: (1) postharvest storage period, categorized as relatively fresh CW (F) or aged CW (A); (2) coconut cultivar, with samples from Wenye No. 2 (N2) or Wenye No. 4 (N4); and (3) maturity, with samples from Wenye No. 2 at 8 months (N2-8) or 10 months (N2-10), and samples from Wenye No. 4 at 6 months (N4-6) or 8 months (N4-8). For the discrimination based on postharvest storage period, samples categorized as F were derived from coconuts stored for 0–7 days, while samples categorized as A were from nuts stored for 15 days or more. Regarding the discriminations for matrices of coconut cultivar and maturity, the samples referred to relatively fresh CW from the corresponding conditions.

The dataset was divided into a training set and an external validation set using a random selection process to minimize potential bias. Specifically, for each day, three out of the five samples of No. 2-8M and No. 4-8M, and two out of the three samples of No. 2-10M and No. 4-6M, were randomly selected and assigned to the training set, while the remaining samples were assigned to the external validation set. This approach ensured that both subsets were representative of the overall dataset and that the model trained on the training set could generalize well to new data.

Spectral data matrix was preprocessed using various techniques, including the first derivative (1st Der), 2nd Der, multiplicative signal correction (MSC), standard normal variate (SNV), row center (RC), Savitzky–Golay (SG), exponentially weighted moving average (EWMA), wavelet compression spectral (WCS), and wavelet denoising spectral (WDS), before modeling. This preprocessing aimed to reduce baseline shift and improve the spectral properties. PCA was performed on the raw and preprocessed spectra to detect trends among samples and identify outliers by evaluating Hotelling’s *T*^2^ range values (at a 5% level of significance) [21]. Subsequently, OPLS-DA was employed to build discrimination models that differentiated CW samples based on postharvest storage time, coconut cultivar, and maturity. The performance of OPLS-DA model was internally validated using a 7-fold cross-validation (CV) approach and assessed for quality using R^2^X_cum_ (the sum of predictive plus orthogonal variation explained in X matrix), R^2^Y_cum_ (total sum of variation explained in Y matrix), and $Q_{cum}^{2}$ (goodness of prediction estimated via CV). External validation was performed to evaluate the overall classification performance of the models, utilizing metrics, such as the percentage of correctly classified observations and AUC (the area under the Receiver Operating Characteristic Curves). The most influential absorption bands in the OPLS-DA classification model were identified using VIP (Variable Importance in Projection) values greater than 1 and *p* values less than 0.05. The reliability of the classifiers was evaluated using CV-ANOVA (analysis of variance testing of cross-validation predictive residuals), considering a *p* value < 0.05 as an indication of a good model [21].

## 3. Results

### 3.1. Distribution and Quantification of Reference Data

**Figure 1:**
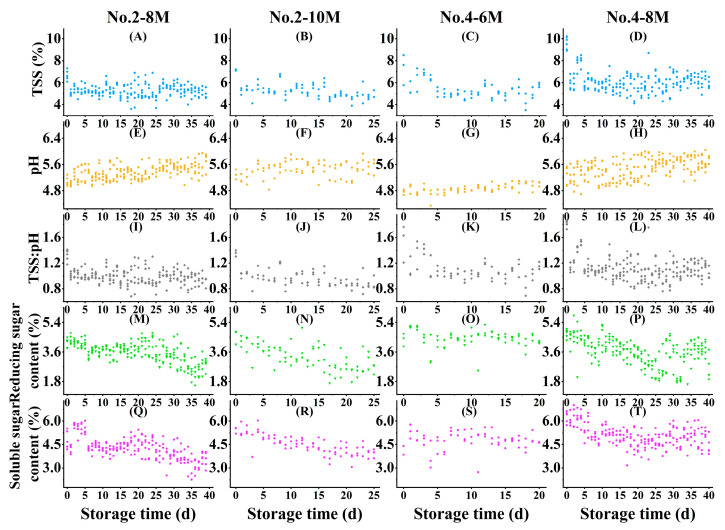
Biochemical properties of CW from postharvest coconuts. Each point represented the mean value of three technical replicates from one biochemical sample. TSS for No. 2-8M (**A**), No. 2-10M (**B**), No. 4-6M (**C**), and No. 4-8M (**D**); pH value for No. 2-8M (**E**), No. 2-10M (**F**), No. 4-6M (**G**), and No. 4-8M (**H**); TSS:pH for No. 2-8M (**I**), No. 2-10M (**J**), No. 4-6M (**K**), and No. 4-8M (**L**); Reducing sugar content for No. 2-8M (**M**), No. 2-10M (**N**), No. 4-6M (**O**), and No. 4-8M (**P**); and soluble sugar content for No. 2-8M (**Q**), No. 2-10M (**R**), No. 4-6M (**S**), and No. 4-8M (**T**).

The distribution and quantification of reference data were analyzed for various attributes, including TSS, pH, TSS/pH, reducing sugar, and soluble sugar. Figure 1 illustrates the behavioral differences between CW samples stored for different times. Due to variations in gene diversity, sampling regions, and physiological differences among coconut samples, the coefficient of variation was high and unstable for the quantified attributes of each individual sampling time (Tables S1–S4). Despite these recorded variations, it was observed that postharvest storage had the effects of raising the value of pH, while decreasing those of TSS, reducing sugar, and soluble sugar content, irrespective of coconut cultivar and maturity. 

### 3.2. Spectral Characteristics

**Figure 2:**
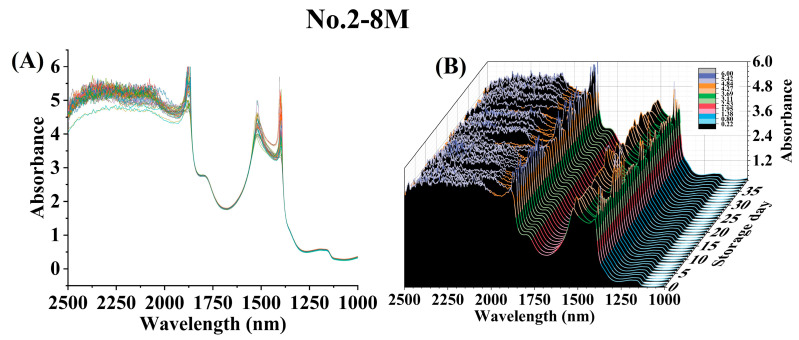
The two-dimensional (**A**) and three-dimensional (**B**) plots of average raw spectra at each postharvest storage time for No. 2-8M.

The average raw spectra (1000–2500 nm) obtained from CW samples at each postharvest storage time for No. 2-8M (Figure 2; Table S5), No. 2-10M (Figure S1; Table S6), No. 4-6M (Figure S2; Table S7), and No. 4-8M (Figure S3; Table S8) were illustrated. Distinct absorption peaks were observed at approximately 1390, 1520, and 1860 nm (Figure 2 and Figures S1–S3), which are typically correlated to the presence of C-H combination, C-H_2_, or C-H (2νCH_3_ and δCH_3_) combination from aliphatic hydrocarbons; N-H (2ν), secondary amine as (R-NH-R) from N-H secondary amine; and C-Cl (7ν), C-Cl from chlorinated hydrocarbons, respectively [22]. Bands with a wide absorption range at 1780–1800 nm are associated with the vibrations produced by O-H stretching and 2 C-O stretching, commonly found in carbohydrate [23]. Carbohydrate-related vibrations were also detected in a wavelength range between 1160–1200 nm, which corresponded to the C-H stretching first overtone [23]. Additionally, despite the noise observed at 1900–2500 nm, bands in this region exhibited intense absorption values (≥4.0), attributed to various combinations of fundamental and overtone vibrations, such as N-H symmetry stretching and amide II from proteins at 2050 nm, O-H stretching and deformation from alcohols at 2073 nm, amide I + amide III from proteins at 2154 nm, O-H stretching and deformation from starch at 2253 nm, and N-H stretching and C-O stretching from proteins at 2292 nm [23]. 

### 3.3. Model Development for Quantitative Analysis

Wavenumbers containing relevant information related to the targeted compound were selected using CARS, VCPA, and ICO algorithms to predict the biochemical parameters using PLSR. The results of this variable selection process are presented in Table 1.

For the reducing sugar content and soluble sugar content, all three methods (CARS, VCPA, and ICO) improved the accuracy of the models, as evidenced by lager RPD and R^2^ values, as well as smaller RMSEF and RMSEP values. Compared to the full-spectrum approach, the RPD values were enhanced by 55.66%, 47.39%, and 55.83% for reducing sugar content, and 32.31%, 34.56%, and 36.51% for soluble sugar content when using CARS, VCPA, and ICO, respectively. Calibration models based on different band selection methods for reducing sugar content performed slightly better than those for soluble sugar content, exhibiting higher RPD values and R^2^ values, as well as smaller RMSEF and RMSEP values. The R^2^ values in the training set ranged from 0.7359 to 0.7682 for reducing sugar content and from 0.6978 to 0.7209 for soluble sugar content. In the external validation set, the R^2^ values were between 0.6999 and 0.7207 for reducing sugar content and between 0.5962 and 0.6197 for soluble sugar content. The achieved RPD values ranged from 1.54 to 1.83, and training set R^2^ values ranged from 0.69 to 0.77, indicating that the NIRS models for reducing sugar and soluble sugar contents provided reliable quantification but lacked sufficient precision [20,24]. However, for TSS, pH, and TSS/pH, the prediction results were unsatisfactory, with R^2^ values below 0.66 and RPD values below 1.5.

### 3.4. Classification of CW Samples Based on Spectral Information

#### 3.4.1. Discrimination using Postharvest Storage Time

The liquid samples from coconuts were categorized into two groups: relatively fresh CW (F), stored for 0–7 days, and aged CW (A), stored for more than 14 (≥15) days. Raw spectral data were divided into two classes (F and A) based on storage period. As shown in Table S9, the training set consisted of 99, 37, 28, and 103 spectra for No. 2-8M, No. 2-10M, No. 4-6M, and No. 4-8M, respectively, while the external validation set included 65, 19, 14, and 68 spectra, respectively. The statistical parameters, such as the number of spectra, R^2^X_cum_, R^2^Y_cum_, $Q_{cum}^{2}$, and *P*_CV-ANOVA,_ were calculated for OPLS-DA calibration, while the classification rate and *p*-value were determined for external validation. Models with the highest total classification rate, along with higher values of R^2^Y_cum_ and $Q_{cum}^{2}$ values were considered as the best pretreatment method for predicting. According to Table S9, the 1st Der pretreatment was the most suitable for discriminating between F and A for No. 2-10M (R^2^Y_cum_ = 0.957, $Q_{cum}^{2}$ = 0.870; total classification rate = 100.00%) and No. 4-6M (R^2^Y_cum_ = 0.992, $Q_{cum}^{2}$ = 0.957; total classification rate = 100.00%). The 2nd Der pretreatment was found to be the most suitable for No. 2-8M (R^2^Y_cum_ = 0.960, $Q_{cum}^{2}$ = 0.747; total classification rate = 95.38%), while EWMA was the best option for No. 4-8M (R^2^Y_cum_ = 0.686, $Q_{cum}^{2}$ = 0.607; total classification rate = 97.06%). 

**Figure 3:**
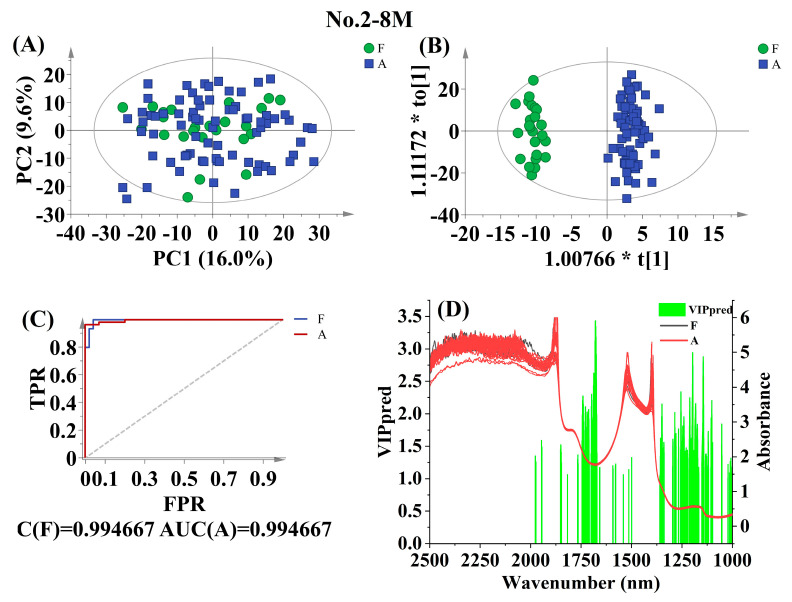
Discrimination results for No. 2-8M according to postharvest storage time. Score scatter plot based on PCA analysis (**A**), Score scatter plot based on OPLS-DA analysis (**B**), AUC prediction value (**C**), and characteristic wavelengths (VIP > 1 and *p* < 0.05) (**D**). The ellipse represents the Hotelling *T*^2^ with 95% confidence interval.

The score scatter plots, AUC, and VIP results from the best-fitted calibration model are illustrated in Figure 3 for No. 2-8M, Figure S4 for No. 2-10M, Figure S5 for No. 4-6M, and Figure S6 for No. 4-8M. However, clear distinctions between F and A were not observed for No. 2-8M and No. 2-10M or No. 4-6M and No. 4-8M, with significant overlap among the spectral points. This could be primarily attributed to the intraclass variability being greater than the among-class variability [25]. To ensure the stability of the monitoring system, outliers beyond the warning limit of Hotelling *T*^2^ with 95% confidence interval were removed. This resulted in the removal of two outliers from Figure 3A, two outliers from Figure S4, one outlier from Figure S5, and five outliers from Figure S6. The remaining data were used as the calibration dataset for the OPLS-DA analysis of No. 2-8M (N = 97), No. 2-10M (N = 35), No. 4-6M (N = 27), and No. 4-8M (N = 98), respectively. The score scatter plots for the OPLS-DA analysis B, Figures S4B–S6B) displayed good interclass variability along the first predictive component, where all F samples corresponded to negative scores and most A samples corresponded to positive scores. However, no tight cluster between F and A was observed along the first orthogonal component, indicating a high interclass variability. Importantly, excellent AUC values (Figure 3C, Figures S4C–S6C) above 0.97 were obtained for No. 2-8M, No. 2-10M, No. 4-6M, and No. 4-8M, with only a few samples experiencing misclassifications. This indicates a satisfactory classification based on postharvest storage time.

The spectral regions that contributed the most to the discrimination between F and A in each calibration are visualized in Figure 3D, Figures S4D–S6D. Among the best calibrations for No. 2-8M and No. 2-10M, common influences were found in the 1591.3–1592.3, 1354.3–1356.5, 1350.6, 1179.8, and 1175.9–1177.0 nm wavelength regions related to N-H from proteins, as well as the 1816.9, 1717.0, 1710.0–1713.5, 1683.5–1684.6, 1679.0–1681.2, 1656.7–1657.8, 1189.9–1192.2, and 1150.5–1151.5 nm wavelength regions related to aromatic hydrocarbons [22]. 

#### 3.4.2. Discrimination by Coconut Cultivar

**Figure 4:**
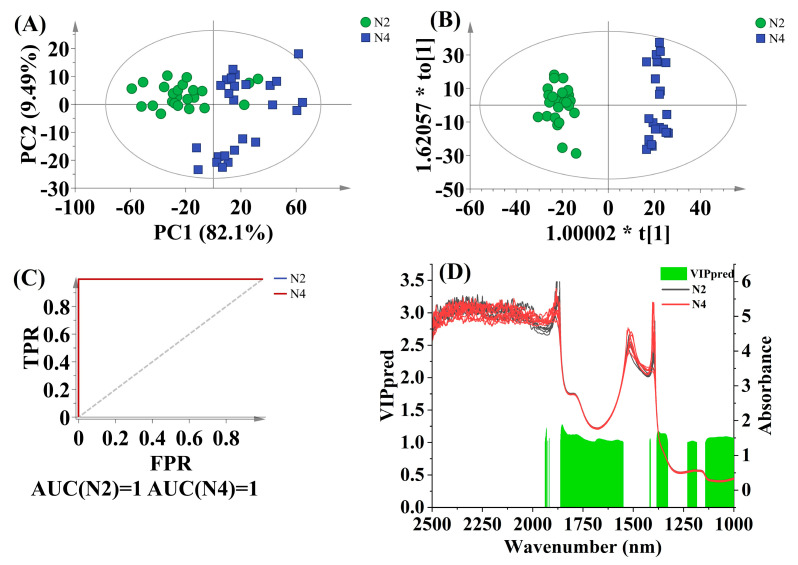
Discrimination results according to coconut cultivar. Score scatter plot based on PCA analysis for N2 (**A**) and OPLS-DA analysis for N4 (**B**). The ellipse represents the Hotelling *T*^2^ with 95% confidence interval. AUC prediction value (**C**) and characteristic wavelengths (VIP > 1 and *p* < 0.05) (**D**).

The discrimination between cultivars of Wenye No. 2 (N2) and Wenye No. 4 (N4) was performed using relatively fresh CW from nuts matured for 8 months, which is considered the most palatable stage in China. A total of 49 spectra were used as the training set, with an additional 31 spectra used for external validation. The statistical analysis of OPLS-DA calibration and external validation for discriminating between N2 and N4 using different pretreatment methods is exhibited in Table S10. The highest total classification rate (100.00%) of the external validation set was achieved with various pretreatment methods, including no treatment, 1st Der, 2nd Der, MSC, SNV, RC, EWMA, and WCS. Among these, the SNV pretreatment model performed the best, exhibiting strong generalization ability, with a higher $Q_{cum}^{2}$ value of 0.916. Figure 4 illustrates the results of the best-fitted PCA, OPLS-DA, AUC analyses, and VIP prediction values for this model.

The PCA analysis of SNV pretreated NIR spectra (Figure 4A) detected one outlier in the CW samples, which was eliminated from the OPLS-DA analysis. This resulted in a total of 48 samples for calibration. Although there is some overlap, a slight separation between N2 and N4 samples can be observed in the score plot (Figure 4A). The score plot along the first predictive component showed two distinct clusters, with positive scores corresponding to N4 samples and negative scores corresponding to N2 samples (Figure 4B). Notably, this model achieved a perfect AUC value of 1 for both N2 and N4 samples (Figure 4C). 

#### 3.4.3. Discrimination by Maturity

**Figure 5:**
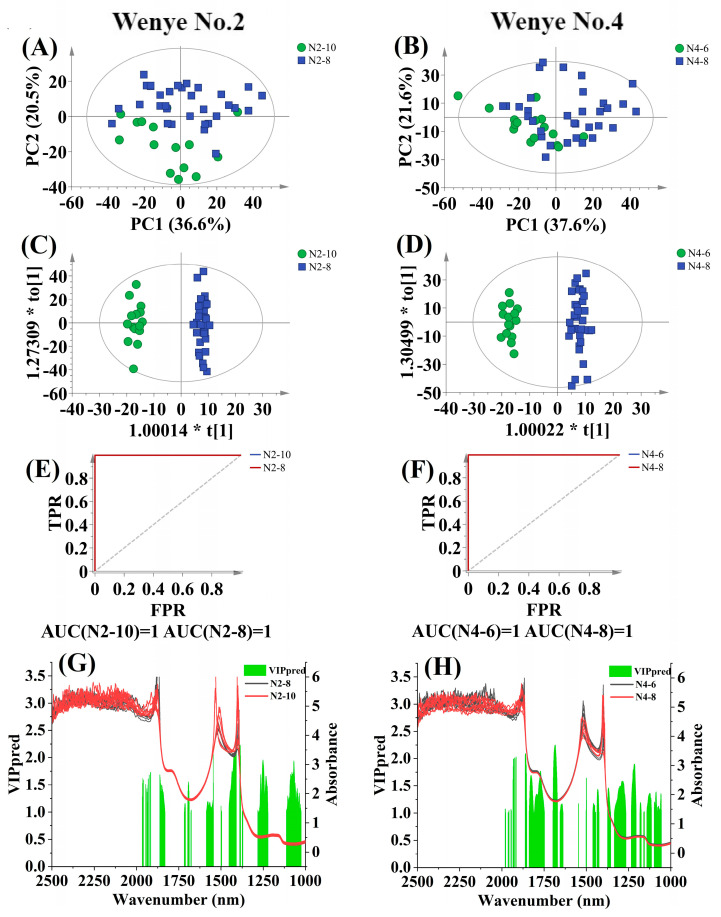
Discrimination results according to maturation stage. Score scatter plot based on PCA analysis for Wenye No. 2 (**A**) and Wenye No. 4 (**B**), and OPLS-DA analysis for Wenye No. 2 (**C**) and Wenye No. 4 (**D**). The ellipse represents the Hotelling *T*^2^ with 95% confidence interval. AUC prediction value for Wenye No. 2 (**E**) and Wenye No. 4 (**F**). Characteristic wavelengths (VIP > 1 and *p* < 0.05) for Wenye No. 2 (**G**) and Wenye No. 4 (**H**).

Table S11 provides statistical information obtained from different preprocessing methods for discriminating CW samples from different maturities. The dataset includes 62 CW samples from Wenye No. 2 at maturity of 8 (N2-8) and 10 (N2-10) months, as well as 65 samples from Wenye No. 4 at 6 (N4-6) and 8 (N4-8) months. According to Table S11, the 1st Der preprocessing method turned out to be the most effective for the discrimination using the maturity mathematical model for both Wenye No. 2 (R^2^Y_cum_ = 0.984, $Q_{cum}^{2}$ = 0.932, total classification rate = 100.00%) and Wenye No. 4 (R^2^Y_cum_ = 0.974, $Q_{cum}^{2}$ = 0.872, total classification rate = 100.00%). These models are further depicted in Figure 5, which includes the score scatter plots of PCA and OPLS-DA analysis, AUC results, and VIP value of the spectral variables. Based on the PCA analysis results shown in Figure 5A,B, no outlier sample points were identified in Wenye No. 2, while one sample point from Wenye No.4 exceeded the ellipse representing Hotelling’s *T*^2^ with a 95% confidence interval. Therefore, the single outlier from Wenye No. 4 was removed to ensure reliable and accurate results before conducting the OPLS-DA models.

For Wenye No. 2, a slight separation between N2-8 and N2-10 samples was observed based on PCA analysis (Figure 5A). However, for Wenye No.4, there was heavy overlap between the natural clustering of CW samples from different maturities, and clear boundaries could not be identified based on PCA analysis (Figure 5B). The score scatter plots of OPLS-DA results in Figure 5C,D indicate a better separation of samples from different maturities. Specifically, the 8-month samples were predominantly distributed on the positive scores of the first predictive component, while CW of N2-10 and N4-6 formed separate clusters on the negative scores. Furthermore, the excellent AUC values of 1 in Figure 5E,F demonstrate the strong performance of both OPLS-DA models.

## 4. Discussion

Water and sugar (5%) are the main constituents of dwarf tender coconut at maturity of 7 months, with low concentrations of acidic substances, such as organic acids and amino acids [26]. In this study, similar results were obtained regarding the sugar content of CW, with reducing sugars ranging from 3.84 to 5.02% and soluble sugars from 3.85 to 6.64% for fresh tender samples, respectively. The dominant soluble sugars found in CW are glucose, sucrose, and fructose [27], and the variation in these sugars may play a vital role in the overall sugar composition of CW. The pH value of fresh tender CW ranged from 4.67 to 5.54, which was influenced by the presence of total non-volatile acids and free organic acids [28]. Additionally, free fatty acids and amino acids identified in CW [4,5,29] may also have contributed to the acidity of samples. Furthermore, the soluble sugar content in No. 4-8M was found to be higher than that in No. 4-6M at the same postharvest storage, whereas no obvious difference was observed between samples from No. 2-8M and No. 2-10M. Similar changes in total soluble sugar content have been reported in CW of ‘Malayan Yellow Dwarf’, where the sugar content increased from 5–6 to 7–8 months old but showed no significant difference from 7–8 to 9–10 months [4]. Moreover, at development stage of 8 months, sugar attributes of reducing sugar and soluble sugar in CW were higher in Wenye No. 4 compared to Wenye No. 2. The pH value of CW from No. 4-6M was lower than that of No. 4-8M at the same postharvest stage, confirming the observation that the pH of CW tended to rise with maturity [6]. These findings suggest the potential of using NIRS combined with chemometrics to discriminate CW samples based on postharvest periods, coconut cultivars, and maturities.

Soluble sugars constitute the major components of CW [30]. In a non-targeted metabolome analysis of CW, 217 metabolites were identified and classified into 11 categories [5]. These components consist of various chemical bonds, including O-H, C-H, N-H, and other functional groups that contain hydrogen. This composition forms the basis for the application of NIRS analysis, as it mainly captures the overtones and vibration combinations of these chemical constituents [31]. Although the spectra of CW from different postharvest storage time, coconut cultivars, and maturities may appear similar, there were still some subtle differences in the absorption bands. However, these differences were not easily discernible via visual inspection alone. Therefore, to effectively analyze these CW samples, chemometric methods were required to extract the relevant information for quantitative and qualitative analysis.

Variable selection is a crucial step in multivariate calibration as it can lead to faster and more cost-effective models, improve prediction accuracy, and enhance the interpretability of the selected variables [17]. The established PLSR models established using CARS, VCPA, and ICO algorithms for reducing sugar and soluble sugar contents demonstrated applicability, with RPD values ranging from 1.54 to 1.83. However, the quantitative models for the TSS, pH, and TSS/pH indices exhibited poor performance, with RPD values below 1.4. One possible explanation for these less favorable prediction results is that these components themselves do not exhibit significant spectral activity within the considered range, as described by Saeys et al. [20]. Another potential reason could be the low content of acids in CW [32], making these parameters more susceptible to noises or interferences and, thereby, challenging their confident prediction. 

In tropical areas, the optimal taste of tender CW is best enjoyed when it is consumed fresh within 7 days of harvesting from the tree [32]. However, if stored for more than 2 weeks, the flavor of the liquid inside the coconut may become altered, resulting in decreased consumer acceptance [33]. For No. 2-8M, the most relevant variables were found to be around 1679 nm, which are associated with C-H methyl groups and carbonyl adjacent as (.C=OCH_3_) related to ketones [22]. Similarly, the relevant variables around 1850 nm exhibited the highest contributions to No. 2-10M, potentially indicating the presence of acid chlorides [22]. The top relevant variables were observed to be around 1437 nm for No. 4-6M, corresponding to the absorption of the first overtone of fundamental stretching band of O-H from phenols and aryl alcohols (Ar-OH) related to phenolic O-H [22]. Likewise, the most relevant variables around 1930 nm showed the highest contributions to No. 4-8M, indicating O-H stretching and HOH bending combinations related to polysaccharides [22]. Overall, the differentiation of CW from different postharvest storage time using NIRS techniques was mainly driven by variations in carbohydrate components. These findings further suggest that the consumption of carbohydrates in CW of postharvest coconut was the main factor contributing to its deterioration.

Quality criteria in CW, such as the water volume per nut ratio, TSS, total sugar, and reducing sugar/total sugar ratio, are important indicators for assessing the suitability of coconut cultivars [30]. The highest VIP values for distinguishing between coconut cultivars are around 1.2, mainly concentrated in the vicinity of 1370 and 1852 nm bands, indicating that aromatic hydrocarbons played a greater role in the identification process of coconut cultivars. The most noticeable difference in CW between Wenye No. 2 and Wenye No. 4 lies in the aroma profile of the liquid inside the kernel. Wenye No. 4, known as an aromatic coconut cultivar in China, is characterized by a pleasant “pandan-like” flavor due to the presence of 2AP [34], whereas Wenye No. 2 is a non-aromatic coconut cultivar. The difference in NIR spectra observed in our study aligns with the flavor diversity between these two coconut varieties.

*Cocos nucifera* L. produces nuts continuously throughout the year, with each nut taking about 12 months to reach full maturity. During the ripening process, CW continues to fill the inside cavity until reaching a development stage of around 11 months. At this point, the volume of CW starts to decrease, and a distinct sound can be heard when the nut is shaken. This characteristic makes it easy for manufacturers to identify nuts older than 11 months, which are typically reserved for breeding purposes and were not considered in this study. Therefore, our focus was on relatively fresh CW from nuts aged 6, 8, and 10 months, with the aim of developing classification models to distinguish between them. For Wenye No. 2, VIP values higher than 2.1 appeared near wavelengths of 1386–1389, 1409–1410, and 1546 nm (Figure 5G). These wavelengths were correlated to C-H combination and O-H (2ν), and O-H related to aliphatic hydrocarbons [22]. Conversely, for Wenye No.4, VIP values higher than 2.1 were located at wavelengths of around 1681–1690 nm (Figure 5H), which were assigned to CONH2 specifically related to peptide β-sheet structures for proteins, C-H methyl, OH associated as ROHCH_3_ for alcohols, and C-H (2ν), ArC-H for aromatic hydrocarbons [22]. These findings suggest that proteins, alcohols, and aromatic hydrocarbons could have important contributions to differentiate CW from maturities of 6, 8, and 10 months.

## 5. Conclusions

NIRS combined with different chemometric methods was utilized as a tool for quantitative analysis of sugar and acid indexes, as well as classification of CW based on postharvest storage time, coconut cultivar, and maturity. The spectral information provided insights into the changes in CW’s chemical constituents under different conditions. Based on our experimental results, the following conclusions can be drawn: (1) PLSR models derived from CW samples achieved better prediction parameters for reducing sugar and soluble sugar contents compared to TSS, pH, and TSS/pH indices. However, the quantitative models for sugar contents were not highly accurate, even with the use of variable selectin algorithms, such as CARS, VCPA, and ICO; (2) NIR spectra combined with the OPLS-DA statistical technique can effectively classify CW samples with up to 100% accuracy based on postharvest storage time, coconut cultivar, and maturity.

To meet the demand of the fresh-eating market, our study focused solely on the analysis of CW from nuts matured for 6–10 months using NIRS. Nuts matured beyond 11 months could be easily distinguished through manual means. Future research will concentrate on developing more precise calibration equations to improve the model’s accuracy in quantifying CW attributes and exploring its practicality in the industry. Furthermore, discrimination models based on sufficient samples originating from diverse cultivars and maturities are needed for commercial purposes.

## Figures and Tables

**Table 1:** Chemometric parameters of PLS calibration protocols to predict biochemical properties via different band selection methods.

Attributes	Number of Collected Samples	Number of Outliers	Variable Selection Methods	Number of Latent Variables	Number of Variables	Training Set		External Validation Set		
						$R_{C}^{2}$	RMSEF	$R_{P}^{2}$	RMSEP	RPD
TSS	544	37	Full-spectrum	6	1131	0.1516	0.7041	−0.0462	0.6578	0.9777
			CARS	6	21	0.1608	0.7003	−0.0473	0.6582	0.9633
			VCPA	8	11	0.4232	0.5806	0.2316	0.5637	1.1077
			ICO	5	128	0.4213	0.5815	0.2395	0.5609	1.1467
pH	544	15	Full-spectrum	1	1131	0.0260	0.3048	0.0265	0.3137	1.0135
			CARS	2	7	0.0610	0.2993	0.0191	0.3149	1.0073
			VCPA	12	12	0.6354	0.1865	0.4776	0.2298	1.2636
			ICO	14	157	0.6570	0.1809	0.4609	0.2334	1.3619
TSS:pH	544	31	Full-spectrum	11	1131	0.2540	0.1225	−0.0876	0.1299	0.9589
			CARS	2	14	0.0894	0.1354	−0.0995	0.1306	0.9280
			VCPA	9	10	0.4584	0.1044	0.1687	0.1136	1.0785
			ICO	14	68	0.3656	0.1130	0.1811	0.1127	1.1051
Reducing sugar content	544	19	Full-spectrum	20	1131	0.4972	0.5436	0.2713	0.6322	1.1715
			CARS	7	31	0.7359	0.3940	0.7207	0.3913	1.8235
			VCPA	10	11	0.7682	0.3691	0.7081	0.4001	1.7267
			ICO	6	93	0.7552	0.3793	0.6999	0.4057	1.8255
Soluble sugar content	544	25	Full-spectrum	20	1131	0.5037	0.5366	0.2635	0.5818	1.1652
			CARS	6	107	0.6978	0.4188	0.5962	0.4307	1.5417
			VCPA	8	11	0.7046	0.4140	0.6197	0.4181	1.5680
			ICO	17	120	0.7209	0.4024	0.6047	0.4262	1.5906

$R_{C}^{2}$: the coefficient of determination for calibration; RMSEF: the root mean squared error of fitting; $R_{P}^{2}$: the coefficient of determination for prediction; RMSEP: the root mean squared error of prediction; RPD: the residual prediction deviation.

## Data Availability

The data presented in this study are available on request from the corresponding author.

## Supplementary Materials

The following supporting information can be downloaded at: https://www.mdpi.com/article/10.3390/foods12122415/s1, Table S1: The mean, standard deviation, range, and coefficient of variation for biochemical traits in the coconut water samples from postharvest nuts for Wenye No. 2 at developmental stage of 8 months; Table S2: The mean, standard deviation, range, and coefficient of variation for biochemical traits in the coconut water samples from postharvest nuts for Wenye No. 2 at developmental stage of 10 months; Table S3: The mean, standard deviation, range, and coefficient of variation for biochemical traits in the coconut water samples from postharvest nuts for Wenye No. 4 at developmental stage of 6 months; Table S4: The mean, standard deviation, range, and coefficient of variation for biochemical traits in the coconut water samples from postharvest nuts for Wenye No. 4 at developmental stage of 8 months; Table S5: The average raw spectra of coconut water samples from postharvest nuts for Wenye No. 2 at developmental stage of 8 months; Table S6: The average raw spectra of coconut water samples from postharvest nuts for Wenye No. 2 at developmental stage of 10 months; Table S7: The average raw spectra of coconut water samples from postharvest nuts for Wenye No. 4 at developmental stage of 6 months; Table S8: The average raw spectra of coconut water samples from postharvest nuts for Wenye No. 4 at developmental stage of 8 months; Table S9: Summary of OPLS-DA calibration statistics and external validation for discrimination by postharvest storage time from matrixes of No. 2-8M, No. 2-10M, No. 4-6M, and No. 4-8M.; Table S10: Summary of OPLS-DA calibration statistics and external validation for discrimination by coconut cultivar; Table S11: Summary of OPLS-DA calibration statistics and external validation for discrimination by maturation stage from matrixes of Wenye No. 2 and Wenye No.4; Figure S1: The two-dimensional (A) and three-dimensional (B) plots of average raw spectra at each postharvest storage time for No. 2-10M; Figure S2: The two-dimensional (A) and three-dimensional (B) plots of average raw spectra at each postharvest storage time for No. 4-6M; Figure S3: The two-dimensional (A) and three-dimensional (B) plots of average raw spectra at each postharvest storage time for No. 4-8M; Figure S4: Discrimination results for No. 2-10M according to postharvest storage time. Score scatter plot based on PCA analysis (A), Score scatter plot based on OPLS-DA analysis (B), AUC prediction value (C), and characteristic wavelengths (VIP > 1 and *p* < 0.05) (D). The ellipse represents the Hotelling *T^2^* with 95% confidence interval; Figure S5: Discrimination results for No. 4-6M according to postharvest storage time. Score scatter plot based on PCA analysis (A), Score scatter plot based on OPLS-DA analysis (B), AUC prediction value (C), and characteristic wavelengths (VIP > 1 and *p* < 0.05) (D). The ellipse represents the Hotelling *T^2^* with 95% confidence interval; Figure S6: Discrimination results for No. 4-8M according to postharvest storage time. Score scatter plot based on PCA analysis (A), Score scatter plot based on OPLS-DA analysis (B), AUC prediction value (C), and characteristic wavelengths (VIP > 1 and *p* < 0.05) (D). The ellipse represents the Hotelling *T^2^* with 95% confidence interval.
