# Supplementary material for: Potential of Near-Infrared Spectroscopy (NIRS) for Efficient Classification Based on Postharvest Storage Time, Cultivar and Maturity in Coconut Water
Source: Foods. 2023 Jun 20;12(12):2415. doi: 10.3390/foods12122415 (PMC10297207; doi:10.3390/foods12122415)
Supplement: Supplementary file 1 [file foods-12-02415-s001.zip › Supplementary Figure.pdf]

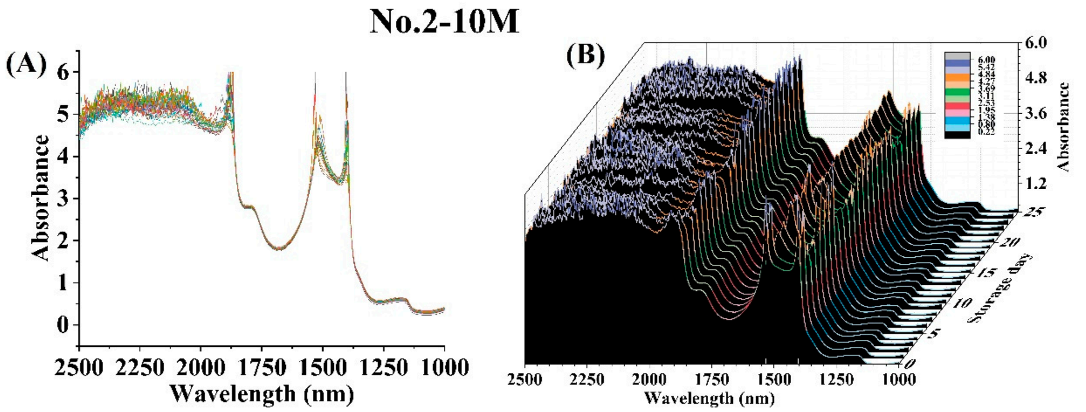

Figure S1. The two-dimensional (**A**) and three-dimensional (**B**) plots of average raw spectra at each postharvest storage time for No.2-10M.

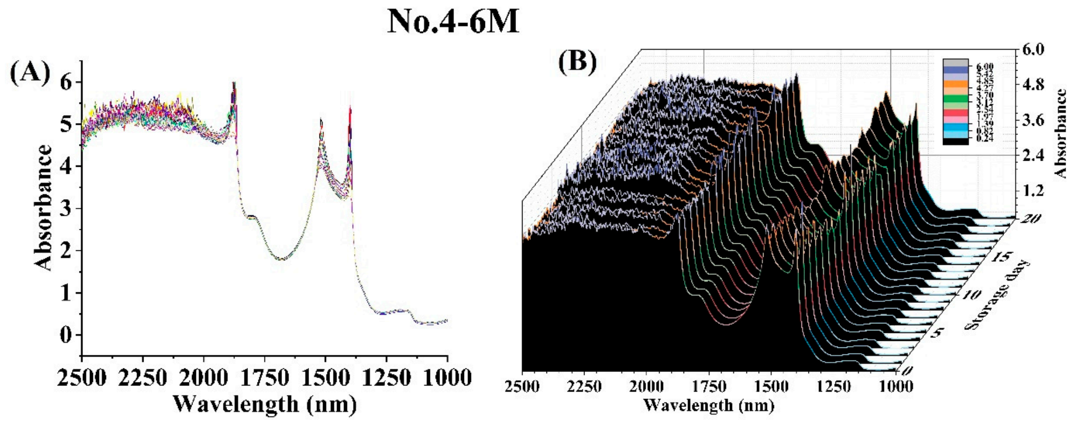

Figure S2. The two-dimensional (**A**) and three-dimensional (**B**) plots of average raw spectra at each postharvest storage time for No.4-6M.

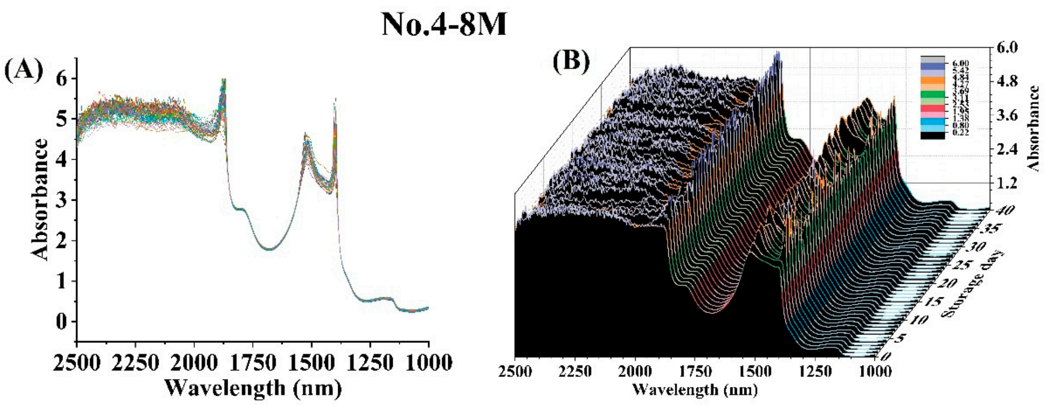

Figure S3. The two-dimensional (**A**) and three-dimensional (**B**) plots of average raw spectra at each postharvest storage time for No.4-8M.

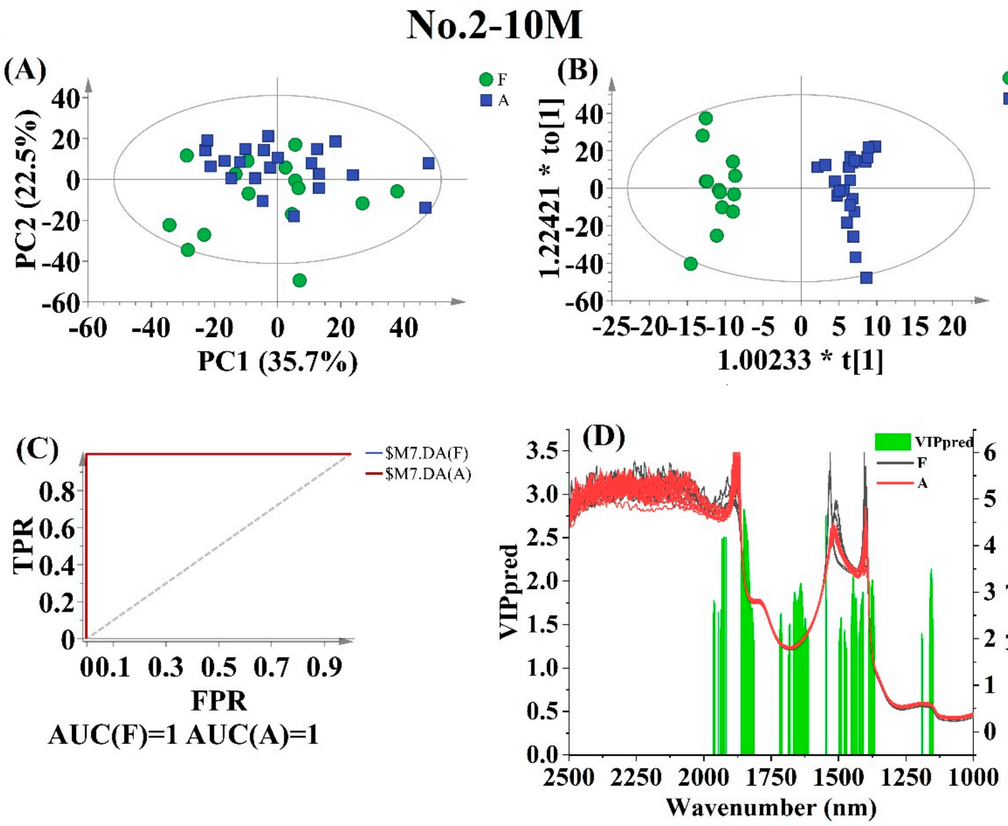

Figure S4. Discrimination results for No.2-10M according to postharvest storage time. Score scatter plot based on PCA analysis (**A**), Score scatter plot based on OPLS-DA analysis (**B**), AUC prediction value (**C**), and characteristic wavelengths (VIP > 1 and $p < 0.05$) (**D**). The ellipse represents the Hotelling T^2 with 95% confidence interval.

No.4-6M

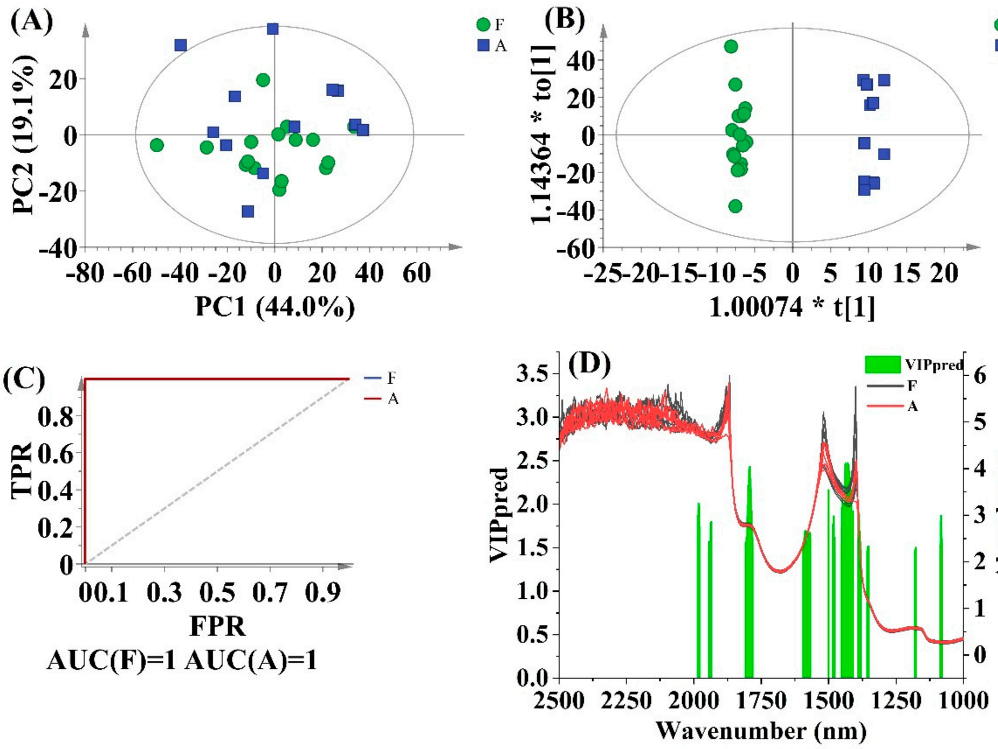

Figure S5. Discrimination results for No.4-6M according to postharvest storage time. Score scatter plot based on PCA analysis (**A**), Score scatter plot based on OPLS-DA analysis (**B**), AUC prediction value (**C**), and characteristic wavelengths (VIP > 1 and $p < 0.05$) (**D**). The ellipse represents the Hotelling T^2 with 95% confidence interval.

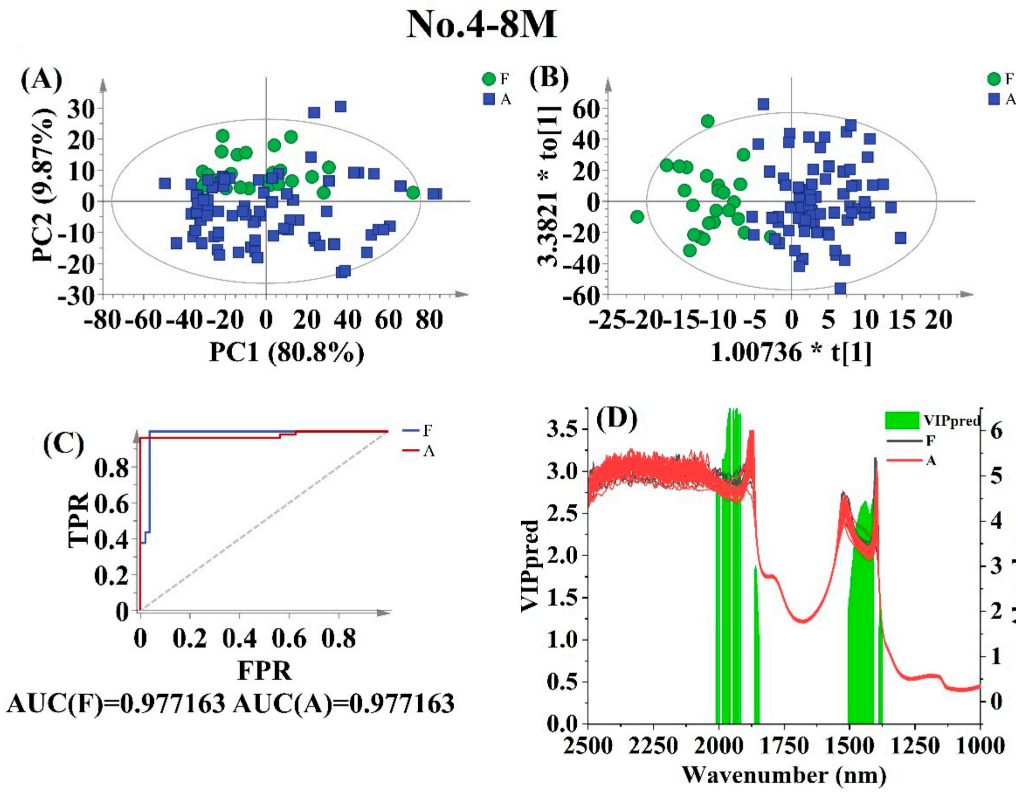

Figure S6. Discrimination results for No.4-8M according to postharvest storage time. Score scatter plot based on PCA analysis (**A**), Score scatter plot based on OPLS-DA analysis (**B**), AUC prediction value (**C**), and characteristic wavelengths (VIP > 1 and $p < 0.05$) (**D**). The ellipse represents the Hotelling T^2 with 95% confidence interval.
